# From the hidden to the obvious: classification of primary and secondary school student suicides using cluster analysis

**DOI:** 10.1186/s12889-022-12983-7

**Published:** 2022-04-09

**Authors:** Anna Wong, Carmen C. S. Lai, Angie K. Y. Shum, Paul S. F. Yip

**Affiliations:** 1 Hong Kong Jockey Club Centre for Suicide Research and Prevention, University of Hong Kong, Hong Kong, China; 2 Department of Social Work and Social Administration, Faculty of Social Sciences, The University of Hong Kong, Hong Kong, China; 3 The HKJC Centre for Suicide Research and Prevention, HKU, The Hong Kong Jockey Club Building for Interdisciplinary Research, 2/F, 5 Sassoon Road, Pokfulam, Hong Kong, China

**Keywords:** Cluster analysis, Student suicide, Risk factor, Stressor, Suicide prevention, Mental health, Warning signs

## Abstract

**Background:**

Suicide is one of the leading causes of death in children and youth. Using a sample of fatal suicides among school-aged students in Hong Kong, this study aimed to demonstrate how the classification of children and adolescent suicides into distinct subgroups using cluster analysis can alert us to the heterogeneous nature of the student suicide population and increase our understanding of multidimensional underlying causes.

**Methods:**

Deaths by suicide of Hong Kong primary and secondary school students occurring between 2013–16 were identified. Reports were acquired from the Coroner’s Court, Police Force, and Education Bureau in Hong Kong. Information about students’ sociodemographic characteristics, suicide circumstances, stressors, and risk factors was extracted and organized for analysis. Based on the indicated stressors (school, family, close relationship, social challenge, finance, risk behaviour, suicide exposure, others) and risk factors (health and mental health, history of self-harm, suicidality, and psychological maladjustment), cluster analysis was conducted to derive distinct profiles of student suicides.

**Results:**

A four-cluster solution was found. Patterns of stressors, risk factors, background characteristics and suicide circumstances within each cluster were examined. Four distinct and meaningful profiles of student suicides were characterised as “school distress”, “hidden”, “family and relationship”, and “numerous issues”.

**Conclusions:**

Findings highlighted the need to approach student suicides in meaningfully differentiated ways. Gathering suicide report data and generating evidence that advances our knowledge of student suicide profiles are important steps towards early identification and intervention.

## Background

Research has shown that distinct subgroups of suicides and suicidal behaviours exist within heterogenous populations. Using the cluster analysis approach, researchers have differentiated greater risk re-attempters from attempters [1], identified fatal attempters with low expressed deliberation [2], and broadened the profiles of suicidal individuals beyond limited demographic characteristics [3]. Furthermore, studies have consistently revealed a two- to four-cluster structure and the existence of an “unlikely” subtype – one that was difficult to identify due to the absence of psychiatric diagnosis, professional help-seeking, or a lack of expressed intent and preparation. Such evidence-based understanding of distinct student subgroups’ characteristics is needed to improve early identification and avoid one-size-fits-all prevention. 

A review of research on children and adolescents’ suicides reveals further important gaps. There are fewer studies on suicides compared to suicidal ideation, attempt, and self-harm. Using Medical Subject Headings (MeSH) to search the PubMed database for suicide studies in 2018–2021, there are 252 studies on completed suicide, 21,537 on non-fatal suicidal behaviour, and 9878 on suicidal ideation. This may be due to researchers’ primary interest in pathways leading to suicidal behaviour and prevention strategies that target these behaviours earlier on. Moreover, studying suicides requires data from multiple governmental departments such as the coroner’s court, which are not accessible to all researchers. Nonetheless, studies of suicidal behaviour and retrospective studies of suicides are both needed for advancing our prediction and prevention strategies [3].

In Hong Kong, there is a widespread concern that academic pressure is a prominent risk factor contributing to student suicide, as confirmed by local research studies which found academic pressure and school distress to be associated with depression and, in turn, suicidality [4, 5]. This concern is in line with international findings, where academic stress was reported in 59.1% of suicides aged 10–14 in Singapore [6], 38% of suicides among children under 18 in the UK [7], and 18.7–22.7% of suicides under age 15 in Turkey [8]. Family factors [9] and mental health conditions such as depression [10] have also received attention, yet few recent studies on local students’ suicides have looked at multiple domains of stressors and risk factors together. To obtain a more comprehensive understanding of their suicides and to identify any underlying pattern or distinct profiles, an investigation into stressors and risk factors in multiple domains among school-aged suicides in Hong Kong is warranted.

This study aimed to extend current understanding of student suicide by identifying distinct profiles of stressors and risk factors of suicide using cluster analysis and to derive implications for suicide detection and prevention.

## Methods

### Study population and data sources

Thirty-five registered cases of suicide among primary and secondary school students (aged 10 to 20) in Hong Kong were included in this study. Cases spanned three consecutive academic years from September 2013 to August 2016. We contacted the Coroner’s Court, Police Force, and Education Bureau requesting copies of official documentation and reports on the registered cases. Sources of evidence included observation and recall by witnesses (e.g. family, peer, teacher, social worker) recorded by the police, official reports on school performance and participation, medical history, post-mortem examination, and death circumstances. Together, they informed our picture of the antecedents and circumstances of their suicides. This study was approved by the Human Research Ethics Committee of the University of Hong Kong.

Sociodemographic characteristics of the deceased were recorded, including the student’s sex, age, local/non-local status (born outside Hong Kong), family income (low income was indicated by receipt of financial assistance under the government’s social welfare scheme), and family intactness (non-intact family was indicated by single parent family, divorce or remarriage in parent’s marital status). Circumstances of suicide, namely the presence of a suicide note or communication on social media prior to suicide and the suicide method, were also documented.

### Analysis

Cluster analysis is a statistical approach that groups data points into subsets such that points within each subset are highly similar to each other and each subset is highly distinct from the others, thus forming clusters. Applying cluster analysis in the study of student suicides categorizes them into distinct subgroups that present as meaningful profiles, each with its own set of patterns and characteristics. Informed by research literature and the available data on risk factors for suicide, 25 indicators were generated and merged into 13 major areas of stressors, health and psychological issues, and suicidality identified as the basic units of a coding framework (See Table 1). All available data were coded according to this standardized checklist of 13 variables, resulting in 35 student records that indicate the presence or absence of any issue under the major areas of concern.

**Table 1:** Background characteristics, suicide circumstance, stressors and risk factors by cluster and between-cluster difference

	**Count (% within Cluster)**				**Count (%)**	**Significance (*p)***
	**Cluster 1** **School distress** (*N* = 14)	**Cluster 2** **Hidden** (*N* = 9)	**Cluster 3** **Family and relationship** (*N* = 7)	**Cluster 4** **Numerous issues** (*N* = 5)	**ALL** (*N* = 35)	**Between-cluster**
**BACKGROUND CHARACTERISTICS**						
**Mean age**	15.7	14.4	15.9	16.4	15.6	0.531
**Sex, male**	7 (50.0%)	8 (88.9%)	5 (71.4%)	3 (60.0%)	23 (65.7%)	0.287
**Intact Family**	11 (78.6%)	6 (66.7%)	7 (100%)	2 (40.0%)	26 (74.3%)	0.113
**Non-local student**	5 (35.7%)	3 (33.3%)	1 (14.3%)	2 (40.0%)	11 (31.4%)	0.752
**Low Income Family** (Received government assistance)	1 (7.1%)	1 (11.1%)	0 (0%)	2 (40.0%)	4 (11.4%)	0.228
**SUICIDE CIRCUMSTANCE**						
**Suicide method**						
**Jumping**^a^	10 (71.4%)	5 (55.6%)	6 (85.7%)	4 (80.0%)	25 (71.4%)	0.628
**Hanging**	2 (14.3%)	3 (33.3%)	0 (0%)	1 (20.0%)	6 (17.1%)	-
**Others** (drowning, suffocation, electrocution)	2 (14.3%)	1 (11.1%)	1 (14.3%)	0 (0%)	4 (11.4%)	-
**Left a suicide note/message on social media**	8 (57.1%)	4 (44.4%)	7 (100%)	2 (40.0%)	21 (60.0%)	0.076
**STRESSORS AND RISK FACTORS**						
**School** (academic, poor relations, SEN, disengagement)	14 (100%)	5 (55.6%)	2 (28.6%)	5 (100%)	26 (74.3%)	0.001
**Family** (poor family relations or conflict, non-conflict issue)	0 (0%)	6 (66.7%)	4 (57.1%)	5 (100%)	15 (42.9%)	0.000
**Close relationship** (poor peer or romantic relations or conflict)	0 (0%)	1 (11.1%)	5 (71.4%)	4 (80.0%)	10 (28.6%)	0.000
**Social challenge** (bullying, dislocation)	0 (0%)	0 (0%)	0 (0%)	4 (80.0%)	4 (11.4%)	0.000
**Finance**	0 (0%)	0 (0%)	0 (0%)	2 (40.0%)	2 (5.7%)	0.017
**Risk behaviour** (smoking, substance abuse, delinquency)	0 (0%)	0 (0%)	1 (14.3%)	2 (40.0%)	3 (8.6%)	0.018
**Suicide exposure**	0 (0%)	0 (0%)	0 (0%)	1 (20.0%)	1 (2.9%)	0.143
**Unspecified issue**	1 (7.1%)	1 (11.1%)	0 (0%)	0 (0%)	2 (5.7%)	1.000
**Physical health issue** (diagnosed or complaint)	7 (50.0%)	1 (11.1%)	2 (28.6%)	3 (60.0%)	13 (37.1%)	0.187
**Self-harm**	2 (14.3%)	0 (0%)	1 (14.3%)	1 (20.0%)	4 (11.4%)	0.536
**Suicidality** (ideation or attempt history)	7 (50.0%)	0 (0%)	7 (100%)	3 (60.0%)	17 (48.6%)	0.000
**Mental health issue** (diagnosed, complaint, or used services)	9 (64.3%)	0 (0%)	2 (28.6%)	3 (60.0%)	14 (40.0%)	0.007
**Psychological maladjustment** (emotional distress, problem coping)	14 (100%)	2 (22.2%)	6 (85.7%)	5 (100%)	27 (77.1%)	0.000

*N* = sample size

^a^Jumping and Not Jumping were compared for between-cluster difference in suicide method

Next, two-step cluster analysis was conducted to derive distinct and meaningful clusters based on the 13 areas of indicated issues. First, Ward’s method was used to estimate the optimal number of clusters, a process of merging the closest, most similar pair of clusters multiple times until a minimum increase in within-cluster variance is found after merging [11, 12]. Next, based on the estimate, several solutions were generated and compared using *K*-means clustering algorithm. Finally, bootstrapping technique was employed to identify the most robust solution, while characteristics of each cluster for that solution were also examined to ensure the classification was meaningful. Analysis of variance and Fisher’s exact test (2-sided) were used as appropriate to test for between-cluster differences in the 13 variables. Observations were made about each cluster, including students’ background characteristics, type of stressors experienced, health and mental health status, history of self-harm and suicidality, and psychological adjustment.

## Results

Three-, four-, and five-cluster solutions were suggested. After testing the solutions in 2000 bootstrap samples, the four-cluster solution was found to be the most robust. The classification was also deemed meaningful after examining characteristics of the four clusters. Fisher’s exact test revealed significant group differences in nine variables, namely school, family, close relationship, social challenge, finance, risk behaviour, suicidality, mental health issue, and psychological maladjustment. Overall, no significant group difference was found in sociodemographic characteristics, suicide circumstance, unspecified issue, suicide exposure, physical health issue, and history of self-harm. The distribution of all variables across the four clusters are summarized in Table 1.

### Sociodemographic characteristics and suicide circumstances

The majority of deceased students were male (65.7%) and the mean age was 15.6. Most came from an intact family (74.3%). About a tenth came from a low-income family (11.4%). Almost a third had a non-local background (31.4%). The most common method was jumping from height (71.4%), followed by hanging (17.1%). Over half of the students left a suicide note or message on social media prior to their suicide (60.0%).

### Stressors and risk factors

In terms of stressors, school issues were the most prevalent, indicated in 74.3% of the students, followed by issues of family (42.9%), close relationship (28.6%), and social challenge (11.4%). Financial issue, unspecified issue, risk behaviour, and suicide exposure were indicated in under 10% of the students.

In terms of health and psychological risk factors, the most frequently reported was psychological maladjustment (77.1%), followed by having a mental illness diagnosis, complaint, or contact with psychological services (40.0%), and having a physical illness diagnosis or complaint (37.1%). Almost half the students had a history of suicidal ideation or attempt (48.6%), about one tenth had self-harmed (11.4%). A significant percentage (37.1%) was documented with neither suicidality nor mental health issues.

### Characteristics of each cluster

#### Cluster 1 “school distress” (*n* = 14)

All students in this cluster were documented with school issues only. Examining school-related issues at the indicator level, 11 students were documented with academic issues, nine with school engagement issues, and four had special educational needs. At the stressor and risk factor level, nine students from this group had mental health issues (64.3%) and seven students had a history of suicidal ideation and/or attempt (50.0%). Three students were exceptions and had no suicidal or mental health issues documented (21.4%). All 14 students from Cluster 1 showed psychological maladjustment, which means that prior to their suicide, all exhibited signs of emotional distress or problem coping. This cluster has the highest proportion of female students (*n* = 7, 50%) compared to other clusters.

#### Cluster 2 “hidden” (*n* = 9)

The majority of students in this cluster were documented with family issues (*n* = 6, 66.7%) and/or school issues (*n* = 5, 55.6%), while one student was also documented with a romantic relationship problem (11.1%). No other known stressors were reported in this cluster. One student, due to not having any indication of a known stressor, was documented with “unspecified issue”. None of the students had a history of self-harm, suicidal ideation/attempt, or mental health issue. Only two students showed psychological maladjustment (22.5%) and another student had a physical health issue (11.1%). This cluster has the highest proportion of male students (*n* = 8, 88.9%).

#### Cluster 3 “family and relationship” (*n* = 7)

Students in this cluster were documented with close relationship (*n* = 5, 71.4%) and/or family issues (*n* = 4, 57.1%). Two students also had additional school issues (28.6%). One student was documented with risk behaviour related to a close relationship issue. All students in this cluster had a history of suicidal ideation/attempt, one student had previously self-harmed (14.3%), two had mental health issue (28.6%), and two had physical health issue (28.6%). Six out of seven students in this cluster were documented with psychological maladjustment (85.7%). Notably, compared with other clusters, Cluster 3 consists of the highest proportion of students from an intact family (100%) and the lowest proportion from a low-income family (0%) and a non-local background (14.3%). All left behind a suicide note or message on social media prior to their suicide (100%).

#### Cluster 4 “numerous issues” (*n* = 5)

Students from this cluster had a median of seven indicated issues per student, the highest of all clusters. Four out of five students in this cluster were documented with all three of school, family, and close relationship issues (80%). The remaining student had school and family issues (20%). All students experienced stressors in other areas, including social challenge (i.e. bullying, dislocation), financial issue, risk behaviour (i.e. substance abuse, delinquency), and exposure to suicide in family or friends. Three out of five students were documented with a history of suicidal ideation/attempt (60.0%), three had a mental health issue (60.0%), and three students had a physical health issue (60.0%). All five students in this cluster were documented with psychological maladjustment (100%). Cluster 4 consists of the highest proportion of students from non-intact (60%) and low-income (40%) families and a non-local background (40%).

## Discussion

To better understand the heterogeneous nature and underlying causes of student suicides, cluster analysis was conducted to identify distinct profiles. A four-cluster solution was found. Patterns of stressors, risk factors, background characteristics and suicide circumstances within each cluster were examined. Four distinct and meaningful profiles of student suicides were characterised as “school distress”, “hidden”, “family and relationship”, and “numerous issues”. In the first cluster, the “school distress” group, school-related issues were accompanied by overwhelmingly adverse states of mental wellness. Although academic issues were most prevalent in this cluster, a considerable proportion of students also experienced interpersonal problems and disengagement at school. Research has shown that school and individual factors may accumulate and interact with one another and impact on students’ perception of school climate, a significant factor influencing suicidal ideation and attempt [13, 14]. Future research may examine these interactions further to understand suicidality of students who are overwhelmed by school problems, since school distress appears to be a prevalent issue in Hong Kong. Moving away from a narrow focus on academic attainment towards a broader focus on student’s wellbeing may mitigate some of the academic stress placed on students [15]. This study also revealed a high proportion of the deceased with special educational needs, reaffirming the necessity to better understand their socio-emotional wellness and to review their suicide risk [16, 17], since the school challenges they face can differ significantly from those affecting students without special education needs.

Students in the second cluster, the “hidden” group, presented an alarming picture since the deceased had no prior history of mental health complaints, disorders or suicidal ideation. This is in line with previous suicide classification studies that consistently identified clusters difficult to detect due to the absence or low occurrence of critical signs such as psychiatric diagnosis, expressed suicide intent, or help-seeking behaviour [1–3]. Cluster two highlights the problem of relying heavily on mental health professionals for risk detection, given the many barriers – stigma, lack of trust, self-reliance – that prevent young people from seeking formal help [18, 19]. Yet, detection of their warning signs in informal settings may have been hindered by their family and/or school issues, while the complexity and ambiguities around suicide communication may also have prevented their true intent from being understood correctly [20]. Therefore, more research is needed to improve help-seeking and understand suicide communication in the student population.

Students in the third cluster, the “family and relationship” group, had primarily family and close relationship issues, although none came from non-intact or low-income families, and all had a history of suicidal ideation and/or attempt. Research findings on close-knitted family relations and suicidality are mixed. While familism, a Latino cultural value that prioritizes the family over the individual, was found to be associated with adolescents’ internalizing behaviour that led to suicidal attempts [21], seeing family responsibility as a reason for living was protective against suicide ideation among adolescents in Hong Kong [4]. Thus, the role of family requires deeper understanding. Finally, all students in cluster three left behind a suicide message on social media, a much higher proportion than other clusters. Some suicide theories view suicidal behaviour as extreme acts of communication intended to convey an important message to significant others [22]. Further research is needed to investigate reasons for young people resorting to suicide for that purpose.

Students in the fourth cluster, the “numerous issues” group, had a distinctly noticeable profile. With the highest proportion of students from non-intact and/or low-income families and the highest total number of indicated issues, they presented a wide range of stressors (including school, family, close relationship, social challenge, financial issue, risk behaviour, exposure to suicide), health and psychological risk factors, and recognisable signs, i.e. a history of self-harm and suicidal behaviour. Research has shown that exposure to multiple levels of risk factors can increase the risk of suicide among adolescents [23]. In contrast with the “hidden” group, risk factors and warning signs are obvious in cluster four. The challenge then becomes how best to prevent suicide among students experiencing difficulties in numerous life domains.

### Limitations and Future Direction

This dataset came from an inquiry commissioned by the government in response to a surge in student suicides leading up to 2016, which alarmed school, health, and social services communities, leaving them unsure of what to do. The suicides of 35 students thus formed a unique set of data collected during a specific timeframe and analysed for a specific purpose. The small sample size of this study was partly determined by its examination of completed suicide rather than attempt or ideation. Also, due to the stigma around suicide and the general consensus that school pressure was prevalent and a risk factor among children and youth, detailed reports and data of student suicide are not normally released without the government’s intervention. This was the first local study that succeeded in collecting comprehensive data on completed student suicides from a range of person- and suicide-specific areas to analyse underlying stressors and risk factors. Although there is no consensus on the optimal sample size and optimal number of variables for conducting cluster analysis, we acknowledge that due to the small size and specificity of our sample, our findings should be interpreted with caution.

Informed by the distinct profiles of suicidal children and adolescents, it became clear that understanding the unique patterns and characteristics of subgroups through further research is crucial for timely detection and tailored intervention. To improve the understanding of suicidality among children and youth, transparency and communication of data and findings are essential. To facilitate this, greater trust and confidence between service providers and research institutes are crucial.

To test and extend current findings, future research should include replication studies and examination of other geographical locations as well as out-of-school children and adolescents. Understanding relationships between influential factors in each subgroup would also significantly advance our knowledge of and strategic response to young people’s suicidality. The existence of the “hidden” group alerted us to challenges around the accessibility and appropriateness of help and support for at-risk students. Since these students are not easily identified, psychoeducation programmes designed to reach all students to increase their mental health literacy and decrease stigma around suicide may be effective in improving their help-seeking behaviour [24, 25]. Training and engaging peer leaders to model positive norms regarding suicide attitude and help-seeking behaviour can be another effective prevention strategy for vulnerable students [26]. The appeal of finding anonymous support in the virtual community has also shown potential for engaging students who are difficult to reach by conventional means [27]. Gatekeeper training may also draw on research of students’ suicidal behaviour as an extreme mode of communication to elucidate the intention and meanings conveyed by those contemplating suicide [22].

## Conclusion

Our study highlighted the heterogenous nature of student suicides and their underlying causes. Emergence of the four distinct clusters reaffirmed the need to understand suicidal students in meaningfully differentiated ways. In response, future research direction relevant to each cluster has been identified and discussed. This study also highlighted a need to develop shared understanding among stakeholders about the importance of gathering detailed suicide report data and generating evidence to inform our knowledge and practice. Promoting open discourse about suicide through public education and research communication may contribute to reduction of stigma, thus facilitate the advancement of research on student suicide. The information on the deceased collected from the Coroner's Court, Police Investigation and the Education Bureau are very useful in formulating  effective suicide prevention measures for suicide prevention [28].

## Data Availability

The datasets used and/or analysed during the current study are available from the corresponding author on reasonable request.
